# An Analysis of the Combination Frequencies of Constituent Medicinal Herbs in Prescriptions for the Treatment of Stroke in Korean Medicine: Determination of a Group of Candidate Prescriptions for Universal Use

**DOI:** 10.1155/2016/2674014

**Published:** 2016-03-20

**Authors:** Byeong Cheol Yun, Seung Bin Pae, Yoo Kyoung Han, Moo Jin Choi, Byung Tae Choi, Hwa Kyoung Shin, Jin Ung Baek

**Affiliations:** ^1^Division of Humanities and Social Medicine, School of Korean Medicine, Pusan National University, Yangsan 626-870, Republic of Korea; ^2^Division of Meridian and Structural Medicine, School of Korean Medicine, Pusan National University, Yangsan 626-870, Republic of Korea

## Abstract

In contrast to Western medicine, which typically prescribes one medicine to treat a specific disease, traditional East Asian medicine uses any one of a large number of different prescriptions (mixtures of medicinal herbs), according to the patient's characteristics. Although this can be considered an advantage, the lack of a universal prescription for a specific disease is considered a drawback of traditional East Asian medicine. The establishment of universally applicable prescriptions for specific diseases is therefore required. As a basic first step in this process, this study aimed to select prescriptions used in the treatment of stroke and, through the analysis of medicinal herb combination frequencies, select a high-frequency medicinal herb combination group for further experimental and clinical research. As a result, we selected some candidates of a medicinal herb combination and 13 candidates of a medicinal herb for the treatment of stroke.

## 1. Introduction

Historically, natural products utilized in traditional medicine have been invaluable for drug development [1, 2]. However, the development of traditional medicines into pharmaceutical products has been challenging; the first step involves the discovery of suitable traditional medicine prescriptions for universal application [3, 4]. While disease symptoms are generally considered similar among patients from the perspective of Western medicine, in traditional East Asian medicine, the selection and administration of one of several tens or hundreds of prescriptions (which are mixture of medicinal herbs) vary according to the individual. This accounts for the diverse variables associated with an individual's external (climate, food, occupation, etc.) and internal (body weight, gender, age, physical strength, etc.) environment. Consequently, while prescriptions which treat the same disease may be combined into a bundle, the nature of the prescriptions in the bundle will be slightly different; this may be considered both a strength and a weakness of traditional East Asian medicine. Combining prescriptions is advantageous because it accounts for variations in individual characteristics; however, it results in lack of universally applicable prescriptions to treat specific diseases.

An attempt to address this involves selecting all of the medicinal herb combinations that exist within each prescription bundle and determining the frequency of these combinations. Despite the loss of individual customizability, selection of the highest frequency medicinal herb combinations may constitute a candidate group for the development of a new prescription for universal application.

Therefore, in this study, after selecting all of the prescriptions for the treatment of stroke (PTSs) recorded in “*Dongeuibogam (dong yi bao gian)*,” a principal piece of Korean medicine literature [5], the frequency of medicinal herb combinations comprising each PTS was analyzed. The aim was to determine a potential candidate group of medicinal herb combinations that can be administered universally for the treatment of stroke.

The rationale for selecting multiple medicinal herb prescriptions, as opposed to single medicinal herbs, is as follows: (1) traditional East Asian medicines are typically available in the form of prescriptions [6]; (2) prescriptions can enhance the effect of the individual constituent medicines and minimize toxicity [7]; and (3) prescriptions are not simply a quantitative addition of the individual medicinal herbs; instead they produce a superior efficacy to single medicines [8, 9].

While several previous studies have analyzed the frequency of medicinal herb combinations for various investigatory purposes [10–12], this present study is the first to use this method in order to develop a universally applicable prescription for the treatment of stroke.

## 2. Materials and Methods

This study comprised three steps. Each step was performed as described in the following paragraphs.

### 2.1. First Step (Figure 1): Establishing a List of PTSs and Constituents of Each Item from the “Pung Chapter” in “Dongeuibogam”

In the first step, after selecting all of the prescriptions recorded in the “*Pung chapter*” (specialized chapter about stroke) in “*Dongeuibogam*,” their indications were analyzed and the medicinal herbs constituting each of the PTSs were selected.

### 2.2. Second Step (Figure 2): Selection of Medicinal Herb Combinations from 92 PTSs in the Order of Frequency

In the second step, the combinations with the highest repeat frequencies were selected as candidates of a medicinal herb combination for the treatment of stroke (CMHCTS), and all medicinal herbs which comprise these combinations were selected as candidates of a medicinal herb for the treatment of stroke (CMHTS). Only the medicinal herbs with doses in the upper 80% cumulative proportion per prescription were included in the CMHCTS. This ensured that only main therapeutic medicinal herbs were selected.

This methodology assumed that the higher the dose within a prescription, the stronger the effect and that the more frequently a medicinal herb is included in prescriptions to treat symptoms, the more important it is [13].

### 2.3. Third Step (Figure 3): Preliminary Evaluation of the Effects of CMHTSs via Analysis of Previous Studies

#### 2.3.1. Selection and Analysis of Previous Studies regarding Effects in Stroke

We searched for 13 CMHTSs in the previous studies and identified relevant studies. Next, studies were specifically divided into* in vitro* studies,* in vivo* studies, clinical studies, and reviews and then analyzed again for research performance status.

#### 2.3.2. Searching the Database

In addition to commonly used scientific databases (such as PubMed, Cochrane, and Scopus), Korean databases (Ndsl, Oasis, and Riss) were used since we were searching specifically for studies related to Korean medicine (KM). The starting period for these study searches was not defined; however, December 31, 2014, was set as the final time point.

#### 2.3.3. Searching Keywords

We used the following terms for the searches: “Scientific names of CMHTS (and Names of herbal medicine of CMHTS) + stroke, cerebral ischemia, ischemia-reperfusion, middle cerebral artery occlusion, hypoxia, oxygen-glucose deprivation, neuroprotection, cerebrovascular protection, anti-neuroinflammation, blood-brain barrier”.

## 3. Results and Discussion

### 3.1. PTSs from the “Pung Chapter” in “Dongeuibogam”

In total, 92 PTSs were selected from the “*Pung chapter*” in “*Dongeuibogam*” and each PTS comprised an average of 7.9 medicinal herbs.

Chinese names followed by the number of constituents are as follows:

 Ba bao hui chun tang (26).
 Ba wei shun qi san (8).
 Ba wu tang jia nan xing ban xia zhi shi zhu li sheng jiang zhi (13).
 Bu huan jin dan (13).
 Chuan xiong shi gao san (8).
 Da qin jiao tang (16).
 Da sheng feng tang (9).
 Dao tan tang (7).
 Di huang yin zi (15).
 Di tan tang (10).
 Ding feng bing zi (9).
 Du shen tang jia zhu li jiang zhi (3).
 Er chen tang (5).
 Er shen dan (10).
 Fang feng tong sheng san (18).
 Huan gu dan (16).
 Huo ming jin dan (15).
 Huo xiang zheng qi san jia nan xing mu xiang fang feng dang gui (17).
 Jia jian dao tan tang (17).
 Jia jian pai feng tang (17).
 Jia jian run zao tang (23).
 Jia jian xu ming tang (13).
 Jia wei da bu tang (23).
 Jia wei jing zhou bai yuan zi (8).
 Jie yu wan (8).
 Jing zhou bai yuan zi (4).
 Li qi qu feng san (17).
 Long xing dan (12).
 Mi chuan shun qi san (18).
 Mu xiang bao ming dan (26).
 Niu huang ding zhi wan (14).
 Niu huang qing xin yuan (30).
 Pai feng tang (14).
 Pi xun ding zi (19).
 Pi yue wan (6).
 Qian zheng san (3).
 Qiang huo yu feng tang (28).
 Qin jiao sheng ma tang (10).
 Qing qi xuan feng san (21).
 Qing shen jie yu tang (19).
 Qing tan shun qi tang (14).
 Qing xin san (9).
 Qing yang tang (10).
 Qu feng chu shi tang (19).
 Qu feng dan (1).
 Qu feng zhi bao dan (26).
 Quan sheng hu gu san (8).
 Ren shen qiang huo san (16).
 Ren shen shun qi san (14).
 San he tang (11).
 San sheng yin (5).
 Shen li tang (16).
 Shen xiang ban xia tang (6).
 Shi quan da bu tang (12).
 Shu feng shun qi tang (22).
 Shu feng shun qi yuan (12).
 Shu feng tang (14).
 Shu jin bao an san (15).
 Si bai dan (20).
 Si jun zi tang jia er chen tang zhu li sheng jiang zhi bai jie zi (12).
 Si jun zi tang jia zhu li sheng jiang zhi (6).
 Si jun zi tang (4).
 Si wu tang jia zhu li sheng jiang zhi fu zi wu tou (8).
 Si wu tang jia zhu li sheng jiang zhi tao ren hong hua bai jie zi (9).
 Si wu tang jia zhu li sheng jiang zhi (6).
 Si wu tang (4).
 Su he xiang yuan (15).
 Su jing yuan (19).
 Tian tai san (19).
 Tian xian gao (4).
 Tie tan yuan (5).
 Tong qi qu feng tang (12).
 Tou bing dan (12).
 Wan jin tang (14).
 Wu long dan (4).
 Wu yao shun qi san (12).
 Xi jiao sheng ma tang (9).
 Xiao tong sheng san (12).
 Xiao xu ming tang (13).
 Xie she bai yuan zi (8).
 Xing fu san (9).
 Xu ming zhu san (15).
 Yang rong tang (20).
 Yi li jin dan (11).
 Yu feng dan (13).
 Yue bi tang (6).
 Yun qi san (10).
 Zheng she san (3).
 Zhi bao dan (11).
 Zhuan she gao (11).
 Zi run tang (10).
 Zi shou jie yu tang (10).

### 3.2. Selection of Medicinal Herb Combinations from 92 PTSs by Frequency Order: Including Only the Medicinal Herbs in the Top 80% Cumulative Percentage for Each Prescription

The following medicinal herb combinations were selected: 82 combinations of one medicinal herb, 581 combinations of two medicinal herbs, 2078 combinations of three medicinal herbs, 5691 combinations of four medicinal herbs, 12,522 combinations of five medicinal herbs, 22,086 combinations of six medicinal herbs, and 31,335 combinations of seven medicinal herbs. By focusing on the top five of each of these (plus ties), selection of the following occurred: six combinations comprising one medicinal herb, five combinations of two medicinal herbs, 13 combinations of three medicinal herbs, seven combinations of four medicinal herbs, six combinations of five medicinal herbs, 19 combinations of six medicinal herbs, and three combinations of seven medicinal herbs. These comprised the CMHCTS with the highest probability of efficacy in the treatment of stroke (Table 1).

### 3.3. Preliminary Evaluation of the Effects of 13 CMHTSs via Analysis of Previous Studies

A total of 1,494 studies of 13 CMHTSs were found; of these, 103 studies were concerned with effects in stroke, resulting in an average of 7.9 publications per candidate herb (Table 2).

### 3.4. Discussion

In this paper, medicinal herbs which have high possibility of stroke treatment effect in KM were selected from “*Dongeuibogam*” by analyzing frequency and effectiveness. Then, analysis of the previous studies has been done.

Look at the possible mechanisms of 13 CMHTSs in Table 2 which shows the final results: (1)* Angelica gigas* Nakai, root: vanillic acid (VA) obtained naturally from the plant* Angelica sinensis* improves spatial learning and memory retention by preventing oxidative stress; (2)* Ostericum koreanum* (Max.) Kitagawa, rhizome:* Ostericum koreanum* has vasodilation effect via change of brain bloodstream; (3)* Arisaema amurense* Maximowicz, rhizome X: none; (4)* Atractylodes japonica* Koidzumi, rhizome:* Atractylodes japonica* Koidzumi prevent the growth inhibition, mitochondrial injury, and apoptosis of neurons induced by hypoxia; (5)* Fraxinus rhynchophylla* Hance, cortex X: none; (6)* Gastrodia elata* Bl., rhizome:* Gastrodia elata* attenuate the hippocampal neuronal damage and decrease necrosis; (7)* Glycyrrhiza uralensis* Fisch., root:* Glycyrrhiza uralensis* Fisch. has neuroprotective efficacy in the postischemic brain via its anti-inflammatory, antiexcitotoxic, and antioxidative effects; (8)* Ligusticum chuanxiong* Hort., rhizome:* Ligusticum chuanxiong* Hort. reduces cerebral infarct through its antioxidative and anti-inflammatory effects; (9)* Paeonia lactiflora* Pallas, root: paeoniflorin may play the role of antagonising cerebral ischemia by adjusting cerebral energy metabolism and nitric oxide formation; (10)* Pinellia ternata* (Thunb.) Breit., rhizome X: none; (11)* Poria cocos* Wolf, sclerotium:* Poria cocos* have neuroprotective effects against the acute restriction of metabolite and oxygen supply in cerebral blood flow; (12)* Rehmannia glutinosa* Liboschitz, root: Catalpol, an iridoid glycoside abundant in the roots of* Rehmannia glutinosa*, exerts the cytoprotective effect on astrocytes by suppressing the production of free radicals and elevating antioxidant capacity; (13)* Scutellaria baicalensis* Georgi, root:* Scutellaria baicalensis* Georgi dramatically reduce the decrease in learning and memory, attenuated neuronal injury, and improved abnormality of energy metabolites.

To sum up, stoke treatment by antioxidative effect and anti-inflammatory effect was mostly common. There were many research papers about neuroprotective effect by energy metabolism and controlling blood circulation as well.

In addition, there are only 4 clinical studies (1 for* Angelica gigas* and 3 for* Ligusticum chuanxiong*) among 103 previous studies. Simply look at the result: (1)* Angelica gigas* Nakai, root:* Angelica* injection has evident therapeutic effect in treating acute cerebral infarction.; (2)* Ligusticum chuanxiong* Hort., rhizome:* Ligusticum chuanxiong* and its effective components improve brain microcirculation through inhibiting thrombus formation and platelet aggregation as well as blood viscosity.

However, in spite of the explanations so far, there could be a few fundamental questions regarding methodology and result of this study since the research method we are using is not general.

First of all, you might ask why classical literature has been used as data instead of clinical data of our times for selecting candidates of medicinal herbs in the first step of method. The answer is that although it is necessary to collect and analyze prescriptions that are frequently used in clinic now for stroke treatment, it is also necessary to discover “a hidden treasure” which was used in the past and might be buried now in classical literature.

Second of all, you might ask why Korean traditional medicinal book was only chosen among many traditional medicinal books. The reason is that Korean traditional medicine has a long tradition but is less studied by researchers compared to Chinese traditional medicine. Thus, we tried to study and discover valuable data from unexplored field.

There are three main reasons for selecting “*Dongeuibogam*” among Korean traditional medicinal books: (1) “*Dongeuibogam*” is the comprehensive summary of all the traditional medicines of Northeast Asia prior to the 17th century, because it is based on rigorous selection of 189 of the major medicinal literature sources of the region [14]; (2) it had a significant impact on not only KM after the 17th century but also on medicinal practices in China and Japan [15]; (3) except for minor content related to superstitions, which were contemporary standards at the time of publication, most of its content is still widely used in modern KM by Korean medical doctors.

Third of all, you may wonder if it is possible to match today's stroke and stroke written in the classical literature. Even though definition of stroke in Korean traditional and Western medicine is slightly different, “*Pung chapter*” specializes in symptom which is the most similar to symptom of today's stroke. Therefore, it is appropriate to match today's stroke and stroke written in the classical literature to select CMHTSs.

Fourth of all, you may wonder why 80% of medicinal herbs in PTS are only included in CMHCTS in the second step of method. In Korean traditional prescription, such as* Zingiber officinale* Rosc. and* Zizyphus jujuba* var.* inermis* Rehder, the so-called “shǐ yào” are added a little for balance of medicinal herbs or to improve digestive functions. These “shǐ yào” do not have major treatment effect but are frequently added in prescriptions, which means that frequently used medicinal herbs in prescription do not mean the herbs are principle ingredients. And therefore, the minor herbs were excluded from CMHCTS and only 80% of medicinal herbs in PTS were included in CMHCTS. The other doubt in the second step of method is that, instead of selecting the most frequently used medicinal herbs in 94 PTSs as CMHTS, why CMHTS is selected after sorting CMHCTS out. The reason is that prescriptions are not simply a quantitative addition of the individual medicinal herbs; instead they produce a superior efficacy to single medicines [8, 9]. Therefore, proposing medicinal herbs of possible combinations instead of single medicines to a clinical researcher could be more useful for follow-up experiment.

Lastly, you may wonder about necessity of third step of method and result of the step, Table 2. In terms of the main purpose of this study (discovering from classical literature), you may find that this step is unnecessary. However, proposing candidates of medicinal herb to clinical researchers by discovering from the classical literature is also the final purpose of this study. Thus, by summarizing previous studies for clinical researchers, it is expected to motivate researchers to conduct follow-up experiments and help to establish research direction using candidates of medicinal herb selected from this research. For the reason above, the third step of the method was carried out and the result of the step is in Table 2.

The fundamental questions discussed above are not only key point but also character of this paper. In conclusion, methodology used in this study is regarded as meaningful challenge to discover “a hidden treasure” for stroke from classical literature. And the result of this study, some CMHCTSs and 13 CMHTSs, will be certainly valuable as fundamental data for experiment and clinical research.

## 4. Conclusions

In the present study, we finally selected some CMHCTSs and 13 CMHTSs from the “*Dongeuibogam*” and reviewed the results of previous studies regarding the effects in stroke. In order to develop a universally applicable PTS, it will be necessary to conduct longer and more complex experiments and clinical trials. However, the CMHCTSs and CMHTSs proposed in this study have the potential to reduce the experimental and developmental time period. Furthermore, this study demonstrates the utilization of text mining for the development of universally applicable prescriptions for a particular disease.

## Figures and Tables

**Figure 1:**
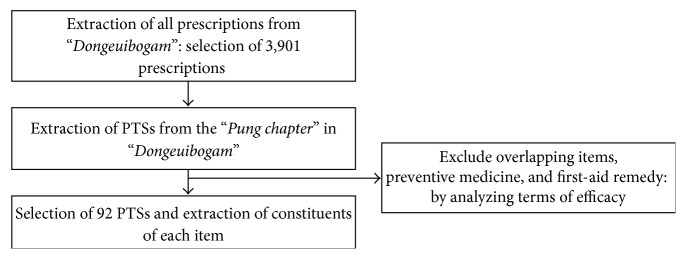
Establishing a list of PTSs and constituents of each item from the “*Pung chapter*” in “*Dongeuibogam.*”

**Figure 2:**
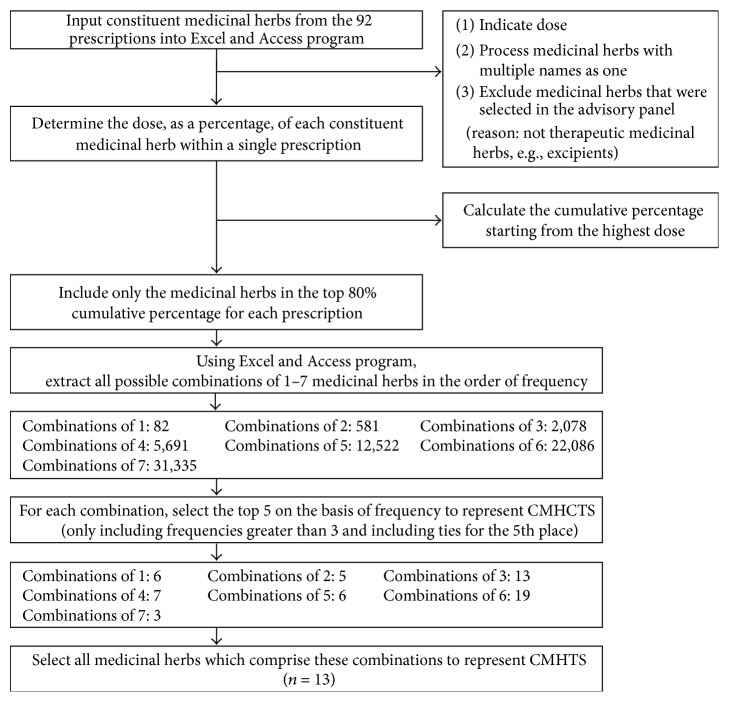
Selection of medicinal herb combinations from 92 PTSs in the order of frequency.

**Figure 3:**
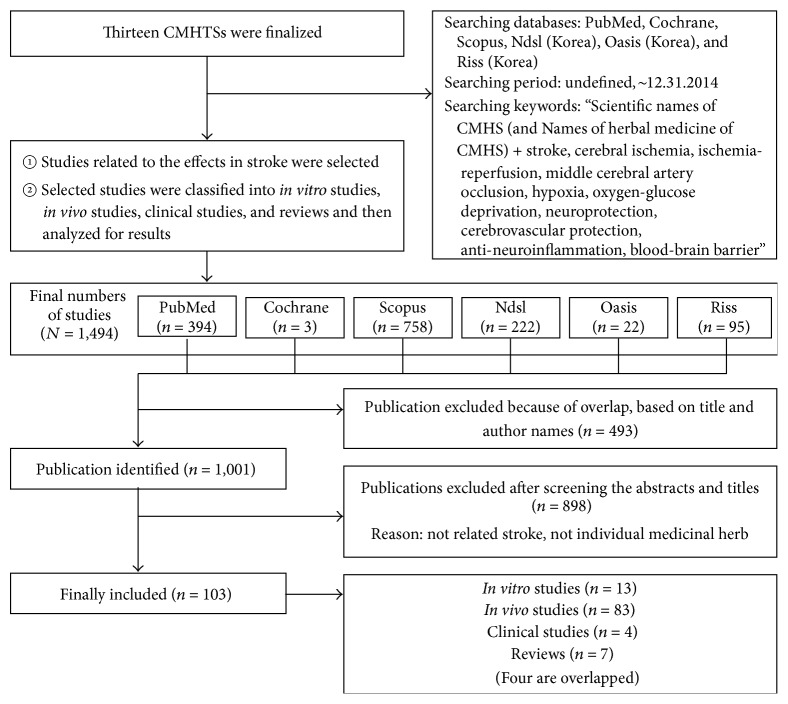
Preliminary evaluation of the effects of 13 CMHTSs via analysis of previous studies.

**Table 1:** Medicinal herb combinations from 92 PTSs in the order of frequency (80%).

Number of constituents	Name of constituents	Frequency
1	(1) *Pinellia ternata* (Thunb.) Breit., rhizome	15
	(2) *Atractylodes japonica* Koidzumi, rhizome	15
	(3) *Arisaema amurense* Maximowicz, rhizome	14
	(4) *Glycyrrhiza uralensis* Fisch., root	12
	(5) *Paeonia lactiflora* Pallas, root	10
	(6) *Poria cocos* Wolf, sclerotium	10

2	(1) *Pinellia ternata* (Thunb.) Breit., rhizome/*Arisaema amurense* Maximowicz, rhizome	9
	(2) *Atractylodes japonica* Koidzumi, rhizome/*Paeonia lactiflora* Pallas, root	8
	(3) *Atractylodes japonica* Koidzumi, rhizome/*Poria cocos* Wolf, sclerotium	8
	(4) *Atractylodes japonica* Koidzumi, rhizome/*Angelica gigas* Nakai, root	7
	(5) *Atractylodes japonica* Koidzumi, rhizome/*Pinellia ternata* (Thunb.) Breit., rhizome	7

3	(1) *Atractylodes japonica* Koidzumi, rhizome/*Paeonia lactiflora* Pallas, root/*Angelica gigas* Nakai, root	7
	(2) *Atractylodes japonica* Koidzumi, rhizome/*Poria cocos* Wolf, sclerotium/*Pinellia ternata* (Thunb.) Breit., rhizome	7
	(3) *Atractylodes japonica* Koidzumi, rhizome/*Paeonia lactiflora* Pallas, root/*Poria cocos* Wolf, sclerotium	6
	(4) *Rehmannia glutinosa* Liboschitz, root/*Paeonia lactiflora* Pallas, root/*Angelica gigas* Nakai, root	5
	(5) *Ligusticum chuanxiong* Hort., rhizome/*Rehmannia glutinosa* Liboschitz, root/*Angelica gigas* Nakai, root	5
	(6) *Ligusticum chuanxiong* Hort., rhizome/*Rehmannia glutinosa* Liboschitz, root/*Paeonia lactiflora* Pallas, root	5
	(7) *Atractylodes japonica* Koidzumi, rhizome/*Poria cocos* Wolf, sclerotium/*Angelica gigas* Nakai, root	5
	(8) *Atractylodes japonica* Koidzumi, rhizome/*Paeonia lactiflora* Pallas, root/*Poria cocos* Wolf, sclerotium	5
	(9) *Atractylodes japonica* Koidzumi, rhizome/*Rehmannia glutinosa* Liboschitz, root/*Angelica gigas* Nakai, root	5
	(10) *Atractylodes japonica* Koidzumi, rhizome/*Rehmannia glutinosa* Liboschitz, root/*Paeonia lactiflora* Pallas, root	5
	(11) *Atractylodes japonica* Koidzumi, rhizome/*Ligusticum chuanxiong* Hort., rhizome/*Rehmannia glutinosa* Liboschitz, root	5
	(12) *Scutellaria baicalensis* Georgi, root/*Atractylodes japonica* Koidzumi, rhizome/*Paeonia lactiflora* Pallas, root	5
	(13) *Scutellaria baicalensis* Georgi, root/*Atractylodes japonica* Koidzumi, rhizome/*Angelica gigas* Nakai, root	5

4	(1) *Atractylodes japonica* Koidzumi, rhizome/*Rehmannia glutinosa* Liboschitz, root/*Paeonia lactiflora* Pallas, root/*Angelica gigas* Nakai, root	5
	(2) *Atractylodes japonica* Koidzumi, rhizome/*Ligusticum chuanxiong* Hort., rhizome/*Rehmannia glutinosa* Liboschitz, root/*Angelica gigas* Nakai, root	5
	(3) *Atractylodes japonica* Koidzumi, rhizome/*Paeonia lactiflora* Pallas, root/*Poria cocos* Wolf, sclerotium/*Pinellia ternata* (Thunb.) Breit., rhizome	5
	(4) *Atractylodes japonica* Koidzumi, rhizome/*Ligusticum chuanxiong* Hort., rhizome/*Rehmannia glutinosa* Liboschitz, root/*Paeonia lactiflora* Pallas, root	5
	(5) *Atractylodes japonica* Koidzumi, rhizome/*Paeonia lactiflora* Pallas, root/*Poria cocos* Wolf, sclerotium/*Angelica gigas* Nakai, root	5
	(6) *Ligusticum chuanxiong* Hort., rhizome/*Rehmannia glutinosa* Liboschitz, root/*Paeonia lactiflora* Pallas, root/*Angelica gigas* Nakai, root	5
	(7) *Scutellaria baicalensis* Georgi, root/*Atractylodes japonica* Koidzumi, rhizome/*Paeonia lactiflora* Pallas, root/*Angelica gigas* Nakai, root	5

5	(1) *Atractylodes japonica* Koidzumi, rhizome/*Ligusticum chuanxiong* Hort., rhizome/*Rehmannia glutinosa* Liboschitz, root/*Paeonia lactiflora* Pallas, root/*Angelica gigas* Nakai, root	5
	(2) *Ligusticum chuanxiong* Hort., rhizome/*Rehmannia glutinosa* Liboschitz, root/*Paeonia lactiflora* Pallas, root/*Poria cocos* Wolf, sclerotium/*Angelica gigas* Nakai, root	4
	(3) *Atractylodes japonica* Koidzumi, rhizome/*Paeonia lactiflora* Pallas, root/*Poria cocos* Wolf, sclerotium/*Pinellia ternata* (Thunb.) Breit., rhizome/*Angelica gigas* Nakai, root	4
	(4) *Atractylodes japonica* Koidzumi, rhizome/*Rehmannia glutinosa* Liboschitz, root/*Paeonia lactiflora* Pallas, root/*Poria cocos* Wolf, sclerotium/*Angelica gigas* Nakai, root	4
	(5) *Atractylodes japonica* Koidzumi, rhizome/*Ligusticum chuanxiong* Hort., rhizome/*Rehmannia glutinosa* Liboschitz, root/*Poria cocos* Wolf, sclerotium/*Angelica gigas* Nakai, root	4
	(6) *Atractylodes japonica* Koidzumi, rhizome/*Ligusticum chuanxiong* Hort., rhizome/*Rehmannia glutinosa* Liboschitz, root/*Paeonia lactiflora* Pallas, root/*Poria cocos* Wolf, sclerotium	4

6	(1) *Atractylodes japonica* Koidzumi, rhizome/*Ligusticum chuanxiong* Hort., rhizome/*Rehmannia glutinosa* Liboschitz, root/*Paeonia lactiflora* Pallas, root/*Poria cocos* Wolf, sclerotium/*Angelica gigas* Nakai, root	4
	(2) *Scutellaria baicalensis* Georgi, root/*Atractylodes japonica* Koidzumi, rhizome/*Paeonia lactiflora* Pallas, root/*Poria cocos* Wolf, sclerotium/*Pinellia ternata* (Thunb.) Breit., rhizome/*Angelica gigas* Nakai, root	3
	(3) *Atractylodes japonica* Koidzumi, rhizome/*Paeonia lactiflora* Pallas, root/*Poria cocos* Wolf, sclerotium/*Pinellia ternata* (Thunb.) Breit., rhizome/*Angelica gigas* Nakai, root/*Ostericum koreanum *(Max.) Kitagawa, rhizome	3
	(4) *Atractylodes japonica* Koidzumi, rhizome/*Rehmannia glutinosa* Liboschitz, root/*Paeonia lactiflora* Pallas, root/*Poria cocos* Wolf, sclerotium/*Pinellia ternata* (Thunb.) Breit., rhizome/*Angelica gigas* Nakai, root	3
	(5) *Atractylodes japonica* Koidzumi, rhizome/*Fraxinus rhynchophylla* Hance, cortex*/Paeonia lactiflora* Pallas, root/*Poria cocos* Wolf, sclerotium/*Pinellia ternata* (Thunb.) Breit., rhizome/*Angelica gigas* Nakai, root	3
	(6) *Atractylodes japonica* Koidzumi, rhizome/*Ligusticum chuanxiong* Hort., rhizome/*Rehmannia glutinosa* Liboschitz, root/*Poria cocos* Wolf, sclerotium/*Pinellia ternata* (Thunb.) Breit., rhizome/*Angelica gigas* Nakai, root	3
	(7) *Atractylodes japonica* Koidzumi, rhizome/*Ligusticum chuanxiong* Hort., rhizome/*Rehmannia glutinosa* Liboschitz, root/*Paeonia lactiflora* Pallas, root/*Pinellia ternata* (Thunb.) Breit., rhizome/*Angelica gigas* Nakai, root	3
	(8) *Atractylodes japonica* Koidzumi, rhizome/*Ligusticum chuanxiong* Hort., rhizome/*Rehmannia glutinosa* Liboschitz, root/*Paeonia lactiflora* Pallas, root/*Poria cocos* Wolf, sclerotium/*Pinellia ternata* (Thunb.) Breit., rhizome	3
	(9) *Ligusticum chuanxiong* Hort., rhizome/*Rehmannia glutinosa* Liboschitz, root/*Paeonia lactiflora* Pallas, root/*Poria cocos* Wolf, sclerotium/*Pinellia ternata* (Thunb.) Breit., rhizome/*Angelica gigas* Nakai, root	3
	(10) *Scutellaria baicalensis* Georgi, root/*Gastrodia elata* Bl., rhizome/*Ligusticum chuanxiong* Hort., rhizome/*Rehmannia glutinosa* Liboschitz, root/*Paeonia lactiflora* Pallas, root/*Angelica gigas* Nakai, root	3
	(11) *Scutellaria baicalensis* Georgi, root/*Atractylodes japonica* Koidzumi, rhizome/*Gastrodia elata* Bl., rhizome/*Ligusticum chuanxiong* Hort., rhizome/*Rehmannia glutinosa* Liboschitz, root/*Paeonia lactiflora* Pallas, root	3
	(12) *Scutellaria baicalensis* Georgi, root/*Atractylodes japonica* Koidzumi, rhizome/*Fraxinus rhynchophylla* Hance, cortex*/Poria cocos* Wolf, sclerotium/*Pinellia ternata* (Thunb.) Breit., rhizome/*Angelica gigas* Nakai, root	3
	(13) *Scutellaria baicalensis* Georgi, root/*Atractylodes japonica* Koidzumi, rhizome/*Fraxinus rhynchophylla* Hance, cortex*/Paeonia lactiflora* Pallas, root/*Pinellia ternata* (Thunb.) Breit., rhizome/*Angelica gigas* Nakai, root	3
	(14) *Scutellaria baicalensis* Georgi, root/*Atractylodes japonica* Koidzumi, rhizome/*Fraxinus rhynchophylla* Hance, cortex*/Paeonia lactiflora* Pallas, root/*Poria cocos* Wolf, sclerotium/*Angelica gigas* Nakai, root	3
	(15) *Scutellaria baicalensis* Georgi, root/*Atractylodes japonica* Koidzumi, rhizome/*Fraxinus rhynchophylla* Hance, cortex*/Paeonia lactiflora* Pallas, root/*Poria cocos* Wolf, sclerotium/*Pinellia ternata* (Thunb.) Breit., rhizome	3
	(16) *Scutellaria baicalensis* Georgi, root/*Atractylodes japonica* Koidzumi, rhizome/*Ligusticum chuanxiong* Hort., rhizome/*Rehmannia glutinosa* Liboschitz, root/*Paeonia lactiflora* Pallas, root/*Angelica gigas* Nakai, root	3
	(17) *Scutellaria baicalensis* Georgi, root/*Atractylodes japonica* Koidzumi, rhizome/*Gastrodia elata* Bl., rhizome/*Rehmannia glutinosa* Liboschitz, root/*Paeonia lactiflora* Pallas, root/*Angelica gigas* Nakai, root	3
	(18) *Scutellaria baicalensis* Georgi, root/*Atractylodes japonica* Koidzumi, rhizome/*Gastrodia elata* Bl., rhizome/*Ligusticum chuanxiong* Hort., rhizome/*Rehmannia glutinosa* Liboschitz, root/*Angelica gigas* Nakai, root	3
	(19) *Atractylodes japonica* Koidzumi, rhizome/*Gastrodia elata* Bl., rhizome/*Ligusticum chuanxiong* Hort., rhizome/*Rehmannia glutinosa* Liboschitz, root/*Paeonia lactiflora* Pallas, root/*Angelica gigas* Nakai, root	3

7	(1) *Scutellaria baicalensis* Georgi, root/*Atractylodes japonica* Koidzumi, rhizome/*Gastrodia elata* Bl., rhizome/*Ligusticum chuanxiong* Hort., rhizome/*Rehmannia glutinosa* Liboschitz, root/*Paeonia lactiflora* Pallas, root/*Angelica gigas* Nakai, root	3
	(2) *Scutellaria baicalensis* Georgi, root/*Atractylodes japonica* Koidzumi, rhizome/*Fraxinus rhynchophylla* Hance, cortex*/Paeonia lactiflora* Pallas, root/*Poria cocos* Wolf, sclerotium/*Pinellia ternata* (Thunb.) Breit., rhizome/*Angelica gigas* Nakai, root	3
	(3) *Atractylodes japonica* Koidzumi, rhizome/*Ligusticum chuanxiong* Hort., rhizome/*Rehmannia glutinosa* Liboschitz, root/*Paeonia lactiflora* Pallas, root/*Poria cocos* Wolf, sclerotium/*Pinellia ternata* (Thunb.) Breit., rhizome/*Angelica gigas* Nakai, root	3

Selecting CMHCTSs as the top 5 on the basis of frequency, only including frequencies greater than 3 and including ties for the 5th place.

**Table 2:** Preliminary evaluation of the effects of 13 CMHTSs in stroke via analysis of the previous studies.

Name of CMHTS	Classification of the study (number)	Source database/main outcome
*Angelica gigas* Nakai, root	VT (2)	(1) P/attenuated A*β*(1-42)-induced neurotoxicity and tau hyperphosphorylation at multiple AD-related sites in a dose-dependent manner [16]
		(2) N and R/inhibited Glu-induced neurotoxicity with IC50 [17]
	VV (24)	(1) S/improved the outcome in rats after cerebral ischemia and reperfusion in terms of neurobehavioral function [18]
		(2) P/improved the habituation memory, decreased AChE, corticosterone, and TNF-*α*, and increased antioxidants [19]
		(3) S/infarct volume of *Angelica* group was significantly decreased [20]
		(4) N/the expression of Ang-2 in the APS group was higher than that in the control group [21]
		(5) P, S, and N/the hyperintense signals and volume in the right cerebrum in *Angelica*-treated group decreased [22]
		(6) N/the expression of VEGF in the *Angelica sinensis* group was higher than that in the other groups [23]
		(7) P and N/increased the gene expression of Flt-1 and Flk-1 [24]
		(8) P, S, and N/reduced cerebral infarct and neurological deficit score and suppressed superoxide radicals in the parenchyma lesion [25]
		(9) P and R/prevented the decrease in the levels of phospho-Akt and phospho-GSK-3*β* [26]
		(10) P, S, and N/prevented neuronal loss, dendrites damage, and neuronal apoptosis in both parietal cortex and hippocampus of 2VO rats [27]
		(11) S and N/reduced brain swelling by 68.62% and 82.08% and significantly improved behavioral deficits [28]
		(12) N/inhibited cyclooxygenase-2 [29]
		(13) P, S, and N/decreased the level of malondialdehyde (MDA) and increased the activities of the antioxidant enzyme glutathione peroxidase (GSH-Px) and superoxide dismutase (SOD) in the ischemic brain tissues [30]
		(14) P and S/reduced malondialdehyde levels and increased superoxide dismutase activity in ischemic brain tissue [31]
		(15) S/decreased the neurologic deficit score and the cerebral infarct volume rate [32]
		(16) P and S/reduced mortality, neurobehavioral deficits, brain edema, BBB permeability, and cerebral vasospasm [33]
		(17) P, S, and N/activated Nrf2/HO-1 pathway [34]
		(18) N and O/GFAP, CD81, and ERK of the brain in rats with cerebral infarction after MCAO were meaningfully decreased [35]
		(19) O and R/induced in infarction areas and volume [36]
		(20) N and O/elevated MCAO-induced decrease in density of neurons and c-Fos immunoreactive cells [37]
		(21) R/had neuroprotective effects via attenuation of COX-2 induction in hippocampus [38]
		(22) N, O, and R/inhibited decreasing the cell viability in ischemia-induced cells [39]
		(23) N, O, and R/reduced infarction volume in ischemic brains of rats, degradation of neuronal cell, BBB permeability, and expression of VEGF protein dose-dependently [40]
		(24) N and R/decreased infarction volume in ischemic brains and inhibited the expression of iNOS, Bax, and caspase-3 [41]
	C (1)	(1) P/decreased infarcted volume [42]
	R (1)	(1) P/increased blood circulation and neuronal metabolism in an MCAO rat model [43]

*Ostericum koreanum *(Max.) Kitagawa, rhizome	VV (1)	(1) O/change of brain bloodstream by preadministered Ds and Dn in cerebral ischemia and blood gas induction by MCAO did not appear [44]

*Arisaema amurense* Maximowicz, rhizome		Not available

*Atractylodes japonica* Koidzumi, rhizome	VT (1)	(1) R/inhibited the hypoxia signaling pathway by reducing HIF-1a expression [45]
	VV (1)	(1) P/prevented growth inhibition, mitochondrial injury, and apoptosis of neurons induced by hypoxia [46]

*Fraxinus rhynchophylla* Hance, cortex		Not available

*Gastrodia elata* Bl., rhizome	VT (2)	(1) S/prevented PC12 cell apoptosis in concentration-dependent manners [47]
		(2) P, S, and N/increased cAMP formation, PKA activity, and phosphorylation of the CREB protein [48]
	VV (13)	(1) P and S/decreased the infarct volume and edema volume and improved the neurological functions after MCAO [49]
		(2) R/reduced infarction area in TTC stain and decreased necrosis in H&E stain [50]
		(3) R/showed lower modified neurological severity score (mNSS) [51]
		(4) P and S/attenuated the hippocampal neuronal damage in the CA1 region in high dose [52]
		(5) P and S/increased the levels of PDI (protein disulfide isomerase) and 1-Cys Prx (peroxiredoxin) transcription [53]
		(6) P, S, and N/increased the expression of Bcl-2 and inhibited the activation of caspase-3 ultimately inhibiting apoptosis [54]
		(7) P and S/expression of PDI, Nrf2, BDNF, GDNF, and MBP genes increased [55]
		(8) N and R/improved the neurological symptoms, reduced infarct volume and cerebral edema, and regulated the expression of CaMKII [56]
		(9) R/decreased infarct size in the brain of GEBs or 4-HBA group [57]
		(10) P and S/prevented hippocampal CA1 cell death following global ischemia [58]
		(11) O and R/had protective effects in the intraperitoneal injection of 1200 mg/kg and 600 mg/kg of Gastrodiae Rhizoma extracts [59]
		(12) O and R/reduced infarct size partly and volume significantly in the MCAO rat brain [60]
		(13) R/showed a significant decrease in infarct size in the ipsilateral brain with the extracts [61]
	R (3)	(1) S/had the greatest neuronal survival after ischemia insult with vanillin-treated animals [62]
		(2) S/protected against neuronal cell damage after transient global ischemia in gerbils [63]
		(3) S/had correlation with stroke by statistics and association analysis [64]

*Glycyrrhiza uralensis* Fisch., root	VV (5)	(1) P, S, and N/had robust neuroprotection in the postischemic brain via anti-inflammatory effect by inhibiting HMGB1 phosphorylation and secretion [65]
		(2) P, S, and N/decreased the focal infarct volume, cerebral histological damage, and apoptosis in MCAO rats [66]
		(3) P, S, and N/the neurological deficits, infarct volume, and the levels of MDA and carbonyl decreased [67]
		(4) P/reduced LDH release from PC12 cells exposed to hypoxic chamber [68]
		(5) P, S, and N/inhibited the increases of brain MDA content and prevented the activities of brain superoxide dismutase (SOD), catalase (CAT), and glutathione peroxidase (GSH-Px) from decline caused by cerebral ischemia-reperfusion [69]

*Ligusticum chuanxiong* Hort., rhizome	VT (6)	(1) N/the levels of tumor necrosis factor-*α* (TNF-*α*), endothelin (ET), and intercellular adhesion molecule-1 (ICAM-1) were lower significantly in the groups treated with volatile oil [70]
		(2) P, S, and N/reduced cerebral infarct and neurological deficit score [25]
		(3) P and R/prevented the decrease in the levels of phospho-Akt and phospho-GSK-3*β* proteins [26]
		(4) P, S, N, and R/decreased the infarct size and behavior deficits score [71]
		(5) N/scores of neurological deficit and infarct volume were lower significantly in the groups treated with volatile oil, and the nitric oxide (NO) and malondialdehyde (MDA) levels were found to be decreased [72]
		(6) O and R/reduced the infarction areas and volume [73]
	C (3)	(1) N/results are healing in 19 cases, obvious effect in 13 cases, availability in 5 cases, and invalidation in 3 cases; healing and obvious effect rate: 80.0% [74] (2) P/the effect of *L. chuanxiong* on the treatment of acute cerebral infarction was superior to low molecular weight dextran [75] (3) P, C, S, and N/improved brain microcirculation through inhibiting thrombus formation and platelet aggregation as well as blood viscosity [76]

*Paeonia lactiflora* Pallas, root	VV (14)	(1) P/ischemia-reperfusion significantly increased AUC values, decreased CL values, and prolonged the terminal half-life of paeoniflorin [77]
		(2) P/prevented reduction of Na(+)-K(+)-ATPase activity, increased NO level, and enhanced NOS activity [78]
		(3) P and S/reduced the infarct volume and alleviated related tongue protrusion (TP) [79]
		(4) S, N, and R/the injuries of ischemia-reperfusion could play an important role in pharmacokinetic process of paeoniflorin in the cortex after intravenous administration of Paeoniae Radix extract [80]
		(5) P/displaced the binding of [3H]NECA to the membrane preparation of rat cerebral cortex in a manner different from its classical agonists [81]
		(6) P and S/reduced protein levels of Ras, MEK, p-MEK, and p-ERK [82]
		(7) P and S/produced delayed protection in the ischemia-injured rats via inhibiting MAPKs/NF-*κ*B mediated peripheral and cerebral inflammatory response [83]
		(8) P/increased cell survival rate and reduced the binding activity of NMDA receptors [84]
		(9) P/inhibited *β*-secretase and apoptosis [85]
		(10) P and S/reduced the cerebral infarction area and the neurodeficit score and reduced lucigenin-CL counts at 2 h period of reperfusion [86]
		(11) P/reduced the decrease of superoxide dismutase (SOD), inhibited the increase of nitric oxide (NO), and lessened the level of malondialdehyde (MDA) and reduced the decrease of lactate dehydrogenase (LDH) in cerebrum remarkably [87]
		(12) P/relieved brain edema, enhanced SOD activity, and lowered MDA concentration in the gerbils and had milder injury of the cells in the hippocampal CA1 region [88]
		(13) P/prolonged gasp time of decapitative mice, lessened cerebral water content, and decreased permeability of cerebral capillary [89]
		(14) P, S, and N/reduced the counts of ED1, IL-1beta, TNF-alpha, and ICAM-1 of microvessels and MPO immunoreactive cells and apoptotic cells [90]
	R (1)	(1) S/showed less potent caspase inhibitory activity [91]

*Pinellia ternata* (Thunb.) Breit., rhizome		Not available

*Poria cocos* Wolf, sclerotium	VV (1)	(1) N/showed neuroprotective effects with EC50 values of 21.6 *μ*g/mL [92]

*Rehmannia glutinosa* Liboschitz, root	VT (1)	(1) P, S, and N/increased astrocyte survival significantly in a concentration-dependent manner [93]
	VV (3)	(1) P and S/enhanced angiogenesis around the infarct of cortex and neurogenesis in the hippocampal dentate gyrus (DG) [94]
		(2) P, S, and N/rescued neurons in hippocampal CA1 subfield and reduced working errors during behavioral testing [95]
		(3) S and N/upregulated GAP-43 protein expression [96]

*Scutellaria baicalensis* Georgi, root	VT (1)	(1) P, S, and N/did not block NMDA-induced neuronal death [97]
	VV (20)	(1) P, S, and N/activated GABAergic signaling and HSP70 and MAPKs cascades in global ischemia [98]
		(2) P, S, and N/performed well in regulating proteins in energy metabolism but had a relatively weak effect in the regulation of proteins in neurogenesis and apoptosis [99]
		(3) P, S, and N/the level of NF-*κ*B p65 was decreased by 73% after baicalin treatment [100]
		(4) P, S, and N/suppressed caspase-3 in ischemic gerbils hippocampus [101]
		(5) P, S, and N/inhibited the formation of 3-nitrotyrosine, reduced infarct size, and attenuated apoptotic cell death, whose effects were similar to FeTMPyP [102]
		(6) P, S, N, and R/inhibited microglial tumor necrosis factor-alpha (TNF-alpha) and nitric oxide production [103]
		(7) P, S, N, and R/the increased contents of MDA and NO and SOD activity and the decreased activity of CAT in the hippocampus and cerebral cortex induced by cerebral ischemia were differently reversed [104]
		(8) P, S, and N/the activities of lactate dehydrogenase, Na(+)-K(+)-ATPase, Ca(2+)-ATPase, and superoxide dismutase were significantly lowered [105]
		(9) P/increased cell survival and inhibited cell apoptosis and excessive production of malondialdehyde [106]
		(10) R/decreased the release of neuron-specific enolase and the production of TBARS [107]
		(11) N/reduced the volume of infarction in the cerebral cortex as well as in the striatum [108]
		(12) P, S, N, and R/reduced the infarct volume, prevented apoptosis in hippocampal cells, attenuated neuronal and blood-brain barrier damage, and upregulated Bcl-2 protein expression [109]
		(13) P and S/reduced brain water content and the permeability of blood vessels, ameliorated ischemia-induced morphology changes in hippocampal microvessels, and downregulated Fas and FasL protein expression [110]
		(14) P, S, N, and R/prolonged gasping time (prolonged ratio: 23.79%) and survival time after carotid artery occlusion and decreased malondialdehyde (MDA) content in damaged brain tissues [111]
		(15) P and S/inhibited PKC(alpha) translocation [112]
		(16) N/protected CA1 hippocampal neurons against 20 min transient forebrain ischemia [113]
		(17) P, S, and N/attenuated neuronal injury and improved abnormality of energy metabolites in rats induced by global ischemia [114]
		(18) P and S/inhibited MMP-9 activity in the hippocampus [115]
		(19) O/CAT and GSH were activated by Scutellaria Radix extract administration [116]
		(20) P, S, and N/reduced the pMCAO- (permanent occlusion of middle cerebral artery-) induced infarct areas in the cerebral cortex as well as in the striatum [117]
	R (2)	(1) S/exerted neuroprotection by inhibiting TNF-*α* [62]
		(2) P/Had antiapoptotic and antiglutamate activity which are the key processes for neuroprotection [118]

^*∗*^PubMed (P), Cochrane (C), Scopus (S), Ndsl (N), Oasis (O), and Riss (R)

^*∗*^ *In vitro* study (VT), *in vivo* study (VV), clinical study (C), and review (R).

## References

[1] Breinbauer R., Manger M., Scheck M., Waldmann H. (2002). Natural product guided compound library development. *Current Medicinal Chemistry*.

[2] Koehn F. E., Carter G. T. (2005). The evolving role of natural products in drug discovery. *Nature Reviews Drug Discovery*.

[3] Corson T. W., Crews C. M. (2007). Molecular understanding and modern application of traditional medicines: triumphs and trials. *Cell*.

[4] Zhang L., Li Y., Guo X. (2014). Text mining of the classical medical literature for medicines that show potential in diabetic nephropathy. *Evidence-Based Complementary and Alternative Medicine*.

[5] Choi M. J., Choi B. T., Shin H. K., Shin B. C., Han Y. K., Baek J. U. (2015). Establishment of a comprehensive list of candidate anti-aging medicinal herb used in Korean medicine by text mining of the classical Korean medical literature, ‘Dongeuibogam’, and preliminary evaluation of the anti-aging effects of these herb. *Evidence-Based Complementary and Alternative Medicine*.

[6] Li S. (2009). Network systems underlying traditional Chinese medicine syndrome and herb formula. *Current Bioinformatics*.

[7] Sucher N. J. (2013). The application of Chinese medicine to novel drug discovery. *Expert Opinion on Drug Discovery*.

[8] Jia W., Gao W.-Y., Yan Y.-Q. (2004). The rediscovery of ancient Chinese herbal formulas. *Phytotherapy Research*.

[9] Scholey A. B., Kennedy D. O. (2002). Acute, dose-dependent cognitive effects of *Ginkgo biloba*, *Panax ginseng* and their combination in healthy young volunteers: differential interactions with cognitive demand. *Human Psychopharmacology: Clinical and Experimental*.

[10] Baek J. U., Lee B. W. (2011). A study on the frequencies of medicinal herb combinations in the prescriptions of ‘Bangyakhappyeon’. *Journal of Korean Medical Classics*.

[11] Baek J. U., Shin S. W., Lee B. W. (2011). A study on the frequencies of medicinal herb combinations in the prescriptions of ‘Wenbingtiaobian’. *Korean Journal of Oriental Medicine*.

[12] Baek J. U., Shin S. W., Lee B. W. (2011). Study on the frequencies of medicinal herb combinations in the prescriptions of ‘Wenrejingwei’. *Korean Journal of Oriental Physiology & Pathology*.

[13] Oh Y. T., Kim S. C., Lee B. W. (2008). Estimation study of the herbal formula's effects by the compositional herbal effects (guideline of the herbal effects intensity). *Journal of Korean Medical Classics*.

[14] Park K. R. (2009). *A bibliographic study on Donguibogam [Doctorate Thesis]*.

[15] Kwon O. M., Cha W. S., Park S. Y., Oh J. H., Ahn S. W. (2011). The appropriation of donguibogam and bencaogangmu and the shaping of distinctive Korean medicine in the late joseon dynasty. *Korean Journal of Oriental Medicine*.

[16] Zhang Z., Zhao R., Qi J., Wen S., Tang Y., Wang D. (2011). Inhibition of glycogen synthase kinase-3*β* by Angelica sinensis extract decreases *β*-amyloid-induced neurotoxicity and tau phosphorylation in cultured cortical neurons. *Journal of Neuroscience Research*.

[17] Cho J. S., Yang C. H., Park C. G. (2000). Inhibition of excitotoxic neuronal cell death by total extracts from oriental medicines used for stroke treatment. *YakhakHoeji*.

[18] Li A., Han L., Han C. C. (2012). Antioxidant and neuroprotective activities of essential oil, isolated from chinese herb pairs of Angelica sinensis and Sophora flavescens. *Journal of Applied Pharmaceutical Science*.

[19] Singh J. C., Kakalij R. M., Kshirsagar R. P., Kumar B. H., Komakula S. S., Diwan P. V. (2014). Cognitive effects of vanillic acid against streptozotocin-induced neurodegeneration in mice. *Pharmaceutical Biology*.

[20] Zhou Q., Liao W., Yang W. (2007). Comparison of effects between angelica sinensis and sodium ferulate on neurological function after reperfusion of focal cerebral ischemia in rats. *Medical Journal of Wuhan University*.

[21] Hu X., Liao W., Yang W., Jiang C., Zhou Q., Cheng M. (2006). Effects of angelica polysaccharide on the expression of angiopoietin after the ischemic brain injury in rats. *Chinese Journal of Rehabilitation Medicine*.

[22] Liao W.-J., Fan M., Yang Y.-H., Yang W.-T., Li L.-Y., Liu M.-L. (2003). Effects of *Angelica sinensis* injection on the neuronal metabolites and blood flow speed within reperfusion following the ischemic cerebral injury in rats. *Chinese Journal of Applied Physiology*.

[23] Meng L., Liao W., Yang W., Zheng C., Jiang C. (2005). Effects of angelica sinensis on the angiogenesis after reperfusion of the ischemia brain injury in rats. *Chinese Journal of Rehabilitation Medicine*.

[24] Zheng C. J., Liao W. J., Fan M., Yang W. T., Meng L. Q. (2006). Effects of *Angelica sinensis* treatment on the expression of flt-1 and flk-1 mRNA after the ischemic brain injury in rats. *Zhongguo Ying Yong Sheng Li Xue Za Zhi*.

[25] Cheng C.-Y., Ho T.-Y., Lee E.-J., Su S.-Y., Tang N.-Y., Hsieh C.-L. (2008). Ferulic acid reduces cerebral infarct through its antioxidative and anti-Inflammatory effects following transient focal cerebral ischemia in rats. *The American Journal of Chinese Medicine*.

[26] Gim S. A., Sung J. H., Shah F. A., Kim M. O., Koh P. O. (2013). Ferulic acid regulates the AKT/GSK-3*β*/CRMP-2 signaling pathway in a middle cerebral artery occlusion animal model. *Laboratory Animal Research*.

[27] Feng Z., Lu Y., Wu X. (2012). Ligustilide alleviates brain damage and improves cognitive function in rats of chronic cerebral hypoperfusion. *Journal of Ethnopharmacology*.

[28] Peng H.-Y., Du J.-R., Zhang G.-Y. (2007). Neuroprotective effect of Z-Ligustilide against permanent focal ischemic damage in rats. *Biological and Pharmaceutical Bulletin*.

[29] Boo Y., Park S., Yu Y. (2003). Neuroprotective Effects of *Angelica gigas* NAKAI on transient focal cerebral ischemia in rats: involvement of antioxidative effects and cyclooxygenase-2 inhibition. *Journal of Cerebral Blood Flow & Metabolism*.

[30] Kuang X., Yao Y., Du J. R., Liu Y. X., Wang C. Y., Qian Z. M. (2006). Neuroprotective role of Z-ligustilide against forebrain ischemic injury in ICR mice. *Brain Research*.

[31] Kuang X., Du J.-R., Liu Y.-X., Zhang G.-Y., Peng H.-Y. (2008). Postischemic administration of Z-Ligustilide ameliorates cognitive dysfunction and brain damage induced by permanent forebrain ischemia in rats. *Pharmacology Biochemistry and Behavior*.

[32] Luo H., Wang X., Su H., Zhu L. (2014). Protection of *Angelica sinensis* volatile oil on focal cerebral ischemia-reperfusion in rats. *Latin American Journal of Pharmacy*.

[33] Chen D., Tang J., Khatibi N. H. (2011). Treatment with Z-ligustilide, a component of *Angelica sinensis*, reduces brain injury after a subarachnoid hemorrhage in rats. *Journal of Pharmacology and Experimental Therapeutics*.

[34] Peng B., Zhao P., Lu Y.-P. (2013). Z-ligustilide activates the Nrf2/HO-1 pathway and protects against cerebral ischemia-reperfusion injury in vivo and in vitro. *Brain Research*.

[35] Song B. K., Jeon Y. C., Kim S. A., Shim A. N., Seong K. M., Lee E. J. (2011). The effect of intravenous injection of the water extract of *Angelica gigas* nakai on gliosis in the middle cerebral artery occlusion rats. *Journal of Pharmacopuncture*.

[36] Jeon Y.-L., Park C.-S., Park C.-G. (2003). An experimental study of effect on brain damage and neuroprotective effect of angelicae gigantis radix extract against cerebral ischemia in rats. *The Korea Journal of Herbology*.

[37] Jeong J. O., Jang W. K., Oh Y. S., Lee S. Y., Park C. S., Park C. G. (2003). The neuroprotective effects of angelicae gigantis radix on focal cerebral ischemia in the rat. *Journal of Korean Oriental Medicine*.

[38] Kim Y. O., Ha N. N., Boo Y. M. (2002). Neuroprotective effect of *Angelica gigas* extracts on the brain ischemia induced by four-vessel occlusion in rats. *The Korea Journal of Herbology*.

[39] Oh T. W., Park K. H., Lee M. Y. (2011). Neuroprotective effects of Angelicae acutilobae Radix water extract against ischemia, reperfusion-induced apoptosis in SK-N-SH neuronal cells. *The Korea Journal of Herbology*.

[40] Park K.-H., Oh T.-W., Park Y.-K. (2012). Neuroprotective effect of the water extract of Angelicae Gigantis Radix Palva in ischemic stroke rats. *The Korea Journal of Herbology*.

[41] Shin Y. J., Park Y. K. (2014). Effects of different parts of *Angelica gigas* Nakai on brain damages and neuronal death in transient middle artery occlusion/reperfusion-induced ischemic rats. *The Korea Journal of Herbology*.

[42] Liu Y.-M., Zhang J.-J., Jiang J. (2004). Observation on clinical effect of Angelica injection in treating acute cerebral infarction. *Zhongguo Zhong xi yi jie he za zhi Zhongguo Zhongxiyi jiehe zazhi*.

[43] Wu Y.-C., Hsieh C.-L. (2011). Pharmacological effects of *Radix Angelica Sinensis (Danggui)* on cerebral infarction. *Chinese Medicine*.

[44] Yun W. S., Kim H. H., Ahn D. K., Rhee J. S., Ham I. H., Choi H. Y. (2004). Effects of angelicaekoreanae radix on the vasomotor responses and focal cerebral ischemic damage by MCAO. *The Korea Journal of Herbology*.

[45] Choi S. W., Chae Y. J., Kim Y. S., Min Y. K., Kim B. T., Lee W. G. Poster session: chemical genomics and drug discovery; SA-61, a polyacetylene compound from rhizome of atractylodes japonica, inhibits the hypoxia signaling pathway by reducing HIF-1a expression.

[46] Hu W.-X., Xiang Q., Wen Z., He D., Wu X.-M., Hu G.-Z. (2014). Neuroprotective effect of Atractylodes macrocephalaon polysaccharides in vitro on neuronal apoptosis induced by hypoxia. *Molecular Medicine Reports*.

[47] Huang N.-K., Chern Y., Fang J.-M., Lin C.-I., Chen W.-P., Lin Y.-L. (2007). Neuroprotective principles from Gastrodia elata. *Journal of Natural Products*.

[48] Tsai C.-F., Huang C.-L., Lin Y.-L., Lee Y.-C., Yang Y.-C., Huang N.-K. (2011). The neuroprotective effects of an extract of Gastrodia elata. *Journal of Ethnopharmacology*.

[49] Zeng X., Zhang Y., Zhang S., Zheng X. (2007). A microdialysis study of effects of gastrodin on neurochemical changes in the ischemic/reperfused rat cerebral hippocampus. *Biological and Pharmaceutical Bulletin*.

[50] Seok P. R., Oh S. J., Choi J. W. Effects of *Gastrodia elata* Blume on the focal brain injury induced by middle cerebral artery occlusion/reperfusion in rats.

[51] Lee S. J. (2009). *Effects of p-hydroxybenzyl alcohol and forced exercise on rat stroke model [M.S. thesis]*.

[52] Kim H.-J., Lee S.-R., Moon K.-D. (2003). Ether fraction of methanol extracts of Gastrodia elata, medicinal herb protects against neuronal cell damage after transient global ischemia in gerbils. *Phytotherapy Research*.

[53] Yu S. J., Kim J. R., Lee C. K. (2005). *Gastrodia elata* blume and an active component, p-hydroxybenzyl alcohol reduce focal ischemic brain injury through antioxidant related gene expressions. *Biological and Pharmaceutical Bulletin*.

[54] Yu S. S., Zhao J., Zheng W. P., Zhao Y. (2010). Neuroprotective effect of 4-hydroxybenzyl alcohol against transient focal cerebral ischemia via anti-apoptosis in rats. *Brain Research*.

[55] Kam K.-Y., Yu S. J., Jeong N. (2011). p-hydroxybenzyl alcohol prevents brain injury and behavioral impairment by activating Nrf2, PDI, and neurotrophic factor genes in a rat model of brain ischemia. *Molecules and Cells*.

[56] Li X. F., Sun Y. K., Zhang W. M., Duan X. H., Dai R., Lin Q. (2011). Protective effect of esterified phenolic compounds from *Gastrodia elata* Blume. on cerebral ischemia-reperfusion injury. *Natural Product Research & Development*.

[57] Seok P. R. (2014). *The protective effects of Gastrodiaelata Blume extracts on the middle cerebral artery occlusion in rats [M.S. thesis]*.

[58] Kim H. J., Hwang I. K., Won M. H. (2007). Vanillin, 4-hydroxybenzyl aldehyde and 4-hydroxybenzyl alcohol prevent hippocampal CA1 cell death following global ischemia. *Brain Research*.

[59] Kim H. C., Ahn D. K. (1999). Neuroprotective effect of gastrodiaerhizoma on global ischemia induced by 4-vessel occlusion in rats. *The Korea Journal of Herbology*.

[60] Younand Y. S., Lee J. S. (2009). Effect of gastrodiaerhizoma on apoptosis in cerebral infarction induced by middle cerebral artery occlusion in rats. *Journal of Oriental Rehabilitation Medicine*.

[61] Lee J. H. (2005). *The inhibitory effects of the extracts, p-hydroxybenzyl alcohol and p-hydroxybenzaldehyde of Gastrodiaelatablume on brain damage in rat ischemic model [Ph.D. thesis]*.

[62] Su S.-Y., Hsieh C.-L. (2011). Anti-inflammatory effects of Chinese medicinal herbs on cerebral ischemia. *Chinese Medicine*.

[63] Behravan E., Razavi B. M., Hosseinzadeh H. (2014). Review of plants and their constituents in the therapy of cerebral ischemia. *Phytotherapy Research*.

[64] Zhang J., Li Y., Chen X., Pan Y., Zhang S., Wang Y. (2014). Systems pharmacology dissection of multi-scale mechanisms of action for herbal medicines in stroke treatment and prevention. *PLoS ONE*.

[65] Kim S.-W., Jin Y., Shin J.-H. (2012). Glycyrrhizic acid affords robust neuroprotection in the postischemic brain via anti-inflammatory effect by inhibiting HMGB1 phosphorylation and secretion. *Neurobiology of Disease*.

[66] Yu X.-Q., Xue C. C., Zhou Z.-W. (2008). In vitro and in vivo neuroprotective effect and mechanisms of glabridin, a major active isoflavan from *Glycyrrhiza glabra* (licorice). *Life Sciences*.

[67] Sun Y.-X., Tang Y., Wu A.-L. (2010). Neuroprotective effect of liquiritin against focal cerebral ischemia/reperfusion in mice via its antioxidant and antiapoptosis properties. *Journal of Asian Natural Products Research*.

[68] Hwang I.-K., Lim S.-S., Choi K.-H. (2006). Neuroprotective effects of roasted licorice, not raw form, on neuronal injury in gerbil hippocampus after transient forebrain ischemia. *Acta Pharmacologica Sinica*.

[69] Zhan C., Yang J. (2006). Protective effects of isoliquiritigenin in transient middle cerebral artery occlusion-induced focal cerebral ischemia in rats. *Pharmacological Research*.

[70] Ling J., Deng W., Zhang J., Li X., Yang F. (2008). Effect of chuangxiong oil on ICAM-1 ,TNF -*α* and ET in focal cerebral ischemia*μ*reperfusion rat. *Pharmacology and Clinics of Chinese Materiamedica*.

[71] Tian J.-W., Fu F.-H., Jiang W.-L., Wang C.-Y., Sun F., Zhang T.-P. (2005). Protective effect of ligusticum chuanxiong phthalides on focai cerebral ischemia in rats and its related mechanism of action. *China Journal of Chinese Materia Medica*.

[72] Ling J., Deng W., Zhang J., Yang F. (2008). The protective effect of Chuanxiong Oil on focal cerebral reperfusion injury in rat. *Pharmacology and Clinics of Chinese Materiamedica*.

[73] Baeg I. S., Park C. S., Park C. G. (2003). The effects of *Cnidium officinale* extract on the ischemic stroke and oxidative neural damage in rats' brain. *The Korea Journal of Herbology*.

[74] Yang J., Bie X., Liu H. (2004). 40 Cases of stroke seqeula treated with chuanxiong extraction by acupoint application. *China Journal of Basic Medicine in Traditional Chinese Medicine*.

[75] Chen D. R. (1992). Comparative study of chuanxiong and dextran 40 in the treatment of acute cerebral infarction. *Zhongguo zhong xi yi jie he za zhi Zhongguo Zhongxiyi jiehe zazhi*.

[76] Chen K. J., Chen K. (1992). Ischemic stroke treated with Ligusticum chuanxiong. *Chinese Medical Journal*.

[77] He X., Xing D., Ding Y., Li Y., Xu L., Du L. (2004). Effects of cerebral ischemia-reperfusion on pharmacokinetic fate of paeoniflorin after intravenous administration of Paeoniae Radix extract in rats. *Journal of Ethnopharmacology*.

[78] Sun R., Lv L.-L., Liu G.-Q. (2006). Effects of paeoniflorin on cerebral energy metabolism, nitric oxide and nitric oxide synthase after cerebral ischemia in mongoliagerbils. *Zhongguo Zhong Yao Za Zhi*.

[79] Xiao L., Wang Y.-Z., Liu J., Luo X.-T., Ye Y., Zhu X.-Z. (2005). Effects of paeoniflorin on the cerebral infarction, behavioral and cognitive impairments at the chronic stage of transient middle cerebral artery occlusion in rats. *Life Sciences*.

[80] Cao C., He X., Wang W., Zhang L., Lin H., Du L. (2006). Kinetic distribution of paeoniflorin in cortex of normal and cerebral ischemia-reperfusion rats after intravenous administration of Paeoniae radix extract. *Biomedical Chromatography*.

[81] Liu D.-Z., Xie K.-Q., Ji X.-Q., Ye Y., Jiang C.-L., Zhu X.-Z. (2005). Neuroprotective effect of paeoniflorin on cerebral ischemic rat by activating adenosine A_1_ receptor in a manner different from its classical agonists. *British Journal of Pharmacology*.

[82] Chen Y.-F., Wu K.-J., Wood W. G. (2013). Paeonia lactiflora extract attenuating cerebral ischemia and arterial intimal hyperplasia is mediated by paeoniflorin via modulation of VSMC migration and Ras/MEK/ERK signaling pathway. *Evidence-based Complementary and Alternative Medicine*.

[83] Guo R.-B., Wang G.-F., Zhao A.-P., Gu J., Sun X.-L., Hu G. (2012). Paeoniflorin protects against ischemia-induced brain damages in rats via inhibiting MAPKs/NF-*κ*B-mediated inflammatory responses. *PLoS ONE*.

[84] Song N.-N., Wu J.-B., Wei X.-B., Guan H.-S., Zhang X.-M. (2009). Paeonol attenuates oxygen-glucose deprivation injury and inhibits NMDA receptor activation of cultured rat hippocampal neurons. *Yao Xue Xue Bao*.

[85] Su S.-Y., Cheng C.-Y., Tsai T.-H., Hsieh C.-L. (2012). Paeonol protects memory after ischemic stroke via inhibiting *β*-secretase and apoptosis. *Evidence-Based Complementary and Alternative Medicine*.

[86] Hsieh C.-L., Cheng C.-Y., Tsai T.-H. (2006). Paeonol reduced cerebral infarction involving the superoxide anion and microglia activation in ischemia-reperfusion injured rats. *Journal of Ethnopharmacology*.

[87] Yang J., Wang J., Feng P., Li Y., Ma C., Xu S. (2000). Protective effect of total paeony glycoside against cerebral ischemia-reperfusion injury in mice. *Zhong yao cai*.

[88] Ma R.-Q., Chen J.-W., Pang J.-X., Lan X.-J., Qiu C.-H. (2005). Protective effects of total paeony glycoside against global cerebral ischemia-reperfusion injury in gerbils. *Di Yi Jun Yi Da XueXueBao*.

[89] Yang J., Wang J., Liu C. (2001). Protective effects of total paeony glycoside on cerebral ischemia mice. *Zhong yao cai*.

[90] Tang N.-Y., Liu C.-H., Hsieh C.-T., Hsieh C.-L. (2010). The anti-inflammatory effect of paeoniflorin on cerebral infarction induced by ischemia-reperfusion injury in sprague-dawley rats. *American Journal of Chinese Medicine*.

[91] Fattorusso R., Frutos S., Sun X., Sucher N. J., Pellecchia M. (2006). Traditional Chinese medicines with caspase-inhibitory activity. *Phytomedicine*.

[92] Lee H. J., Koo U., Lee H. J., Lee D. H., Mar W. C. (2009). Neuroprotective effects of some plant extracts against oxygen-glucose deprivation (OGD)-induced oxidative cell death on neuronal cell. *Korean Journal of Medicinal Crop Science*.

[93] Li Y., Bao Y., Jiang B. (2008). Catalpol protects primary cultured astrocytes from in vitro ischemia-induced damage. *International Journal of Developmental Neuroscience*.

[94] Liu Y., Xue Q., Li X. (2014). Amelioration of stroke-induced neurological deficiency by lyophilized powder of catapol and puerarin. *International Journal of Biological Sciences*.

[95] Li D.-Q., Li Y., Liu Y., Bao Y.-M., Hu B., An L.-J. (2005). Catalpol prevents the loss of CA1 hippocampal neurons and reduces working errors in gerbils after ischemia-reperfusion injury. *Toxicon*.

[96] Zhu H.-F., Wan D., Luo Y., Xie P., Xu X.-Y. (2007). Catalpol up-regulated GAP-43 protein expression and improved behavior outcome of focal cerebral ischemia rats. *Chinese Pharmacological Bulletin*.

[97] Sun X., Chan L. N., Gong X., Sucher N. J. (2003). N-methyl-D-aspartate receptor antagonist activity in traditional Chinese stroke medicines. *Neuro-Signals*.

[98] Dai J., Chen L., Qiu Y.-M. (2013). Activations of GABAergic signaling, HSP70 and MAPK cascades are involved in baicalin's neuroprotection against gerbil global ischemia/reperfusion injury. *Brain Research Bulletin*.

[99] Zhang Z., Wu R., Li P. (2009). Baicalin administration is effective in positive regulation of twenty-four ischemia/reperfusion-related proteins identified by a proteomic study. *Neurochemistry International*.

[100] Xue X., Qu X.-J., Yang Y. (2010). Baicalin attenuates focal cerebral ischemic reperfusion injury through inhibition of nuclear factor *κ*B p65 activation. *Biochemical and Biophysical Research Communications*.

[101] Cao Y., Mao X., Sun C. (2011). Baicalin attenuates global cerebral ischemia/reperfusion injury in gerbils via anti-oxidative and anti-apoptotic pathways. *Brain Research Bulletin*.

[102] Xu M., Chen X., Gu Y. (2013). Baicalin can scavenge peroxynitrite and ameliorate endogenous peroxynitrite-mediated neurotoxicity in cerebral ischemia-reperfusion injury. *Journal of Ethnopharmacology*.

[103] Kim Y. O., Leem K., Park J. (2001). Cytoprotective effect of Scutellaria baicalensis in CA1 hippocampal neurons of rats after global cerebral ischemia. *Journal of Ethnopharmacology*.

[104] Shang Y.-Z., Miao H., Cheng J.-J., Qi J.-M. (2006). Effects of amelioration of total flavonoids from stems and leaves of *Scutellaria baicalensis Georgi* on cognitive deficits, neuronal damage and free radicals disorder induced by cerebral ischemia in rats. *Biological & Pharmaceutical Bulletin*.

[105] Shang Y. Z., Zhang H., Cheng J. J. (2013). Flavonoids from scutellaria baicalensis georgi are effective to treat cerebral ischemia/reperfusion. *Neural Regeneration Research*.

[106] Shang Y., Miao G., Zhao H. (2014). Mechanisms underlying attenuation of apoptosis of cortical neurons in the hypoxic brain by flavonoids from the stems and leaves of *Scutellaria baicalensis* Georgi. *Neural Regeneration Research*.

[107] Kwon H. Y. (2011). *Neuroprotective effect of baicalein in experimental ischemia-reperfusion brain injury [Ph.D. thesis]*.

[108] Cho J. S., Lee H. K. (2002). Neuroprotective effect of woganin in a rodent model of permanent focal cerebral ischemia. *Proceedings of the Convention of the Pharmaceutical Society of Korea*.

[109] Zhao S. M., Kong W., Zhang S. F., Chen M., Zheng X. Y., Kong X. Y. (2013). Pretreatment with scutellaria baicalensis stem-leaf total flavonoid prevents cerebral ischemia-reperfusion injury. *Neural Regeneration Research*.

[110] Kong X. Y., Kong W., Miao G. X. (2014). Pretreatment with scutellaria baicalensis stem-leaf total flavonoid protects against cerebral ischemia/ reperfusion injury in hippocampal neurons. *Neural Regeneration Research*.

[111] Zhang Y., Wang X., Wang X. (2006). Protective effect of flavonoids from *Scutellaria baicalensis* Georgi on cerebral ischemia injury. *Journal of Ethnopharmacology*.

[112] Liu L.-Y., Wei E.-Q., Zhao Y.-M. (2005). Protective effects of baicalin on oxygen/glucose deprivation- and NMDA-induced injuries in rat hippocampal slices. *Journal of Pharmacy and Pharmacology*.

[113] Kim Y. O., Park J. Y., Joe K. H. (2000). *Scutellaria baicalensis* protects CA1 hippocampal neurons after global cerebral ischemia in rats. *Proceedings of the Convention of the Pharmaceutical Society of Korea*.

[114] Shang Y., Cheng J., Qi J., Miao H. (2005). Scutellaria flavonoid reduced memory dysfunction and neuronal injury caused by permanent global ischemia in rats. *Pharmacology Biochemistry and Behavior*.

[115] Lee J.-H., Lee S.-R. (2012). The effect of baicalein on hippocampal neuronal damage and metalloproteinase activity following transient global cerebral ischaemia. *Phytotherapy Research*.

[116] Park J. E., Kim Y. K. (2009). The effect of scutellariae radix on ischemia induced brain injury in rats. *The Korean Journal of Joongpoong*.

[117] Cho J. S., Lee H.-K. (2004). Wogonin inhibits ischemic brain injury in a rat model of permanent middle cerebral artery occlusion. *Biological and Pharmaceutical Bulletin*.

[118] Gaire B. P., Moon S.-K., Kim H. (2014). Scutellaria baicalensis in stroke management: nature's blessing in traditional Eastern medicine. *Chinese Journal of Integrative Medicine*.

